# Fast and Effective Photodynamic Inactivation of Multiresistant Bacteria by Cationic Riboflavin Derivatives

**DOI:** 10.1371/journal.pone.0111792

**Published:** 2014-12-03

**Authors:** Tim Maisch, Anja Eichner, Andreas Späth, Anita Gollmer, Burkhard König, Johannes Regensburger, Wolfgang Bäumler

**Affiliations:** 1 Department of Dermatology, University Hospital Regensburg, Regensburg, Germany; 2 Institute of Organic Chemistry, University of Regensburg, Regensburg, Germany; University of Cambridge, United Kingdom

## Abstract

Photodynamic inactivation of bacteria (PIB) proves to be an additional method to kill pathogenic bacteria. PIB requires photosensitizer molecules that effectively generate reactive oxygen species like singlet oxygen when exposed to visible light. To allow a broad application in medicine, photosensitizers should be safe when applied in humans. Substances like vitamin B2, which are most likely safe, are known to produce singlet oxygen upon irradiation. In the present study, we added positive charges to flavin derivatives to enable attachment of these molecules to the negatively charged surface of bacteria. Two of the synthesized flavin derivatives showed a high quantum yield of singlet oxygen of approximately 75%. Multidrug resistant bacteria like MRSA (Methicillin resistant *Staphylococcus aureus*), EHEC (enterohemorrhagic *Escherichia coli*), *Pseudomonas aeruginosa*, and *Acinetobacter baumannii* were incubated with these flavin derivatives *in vitro* and were subsequently irradiated with visible light for seconds only. Singlet oxygen production in bacteria was proved by detecting its luminescence at 1270 nm. After irradiation, the number of viable bacteria decreased up to 6 log_10_ steps depending on the concentration of the flavin derivatives and the light dosimetry. The bactericidal effect of PIB was independent of the bacterial type and the corresponding antibiotic resistance pattern. In contrast, the photosensitizer concentration and light parameters used for bacteria killing did not affect cell viability of human keratinocytes (therapeutic window). Multiresistant bacteria can be safely and effectively killed by a combination of modified vitamin B2 molecules, oxygen and visible light, whereas normal skin cells survive. Further work will include these new photosensitizers for topical application to decolonize bacteria from skin and mucosa.

## Introduction

Bacteria are champions of evolution and a few microbes have adapted to a point where they pose serious clinical challenges for humans [1], [2]. Among the Gram-positive organisms, methicillin resistant *Staphylococcus aureus* (MRSA) causes major clinical problems. Thus, several compounds have been developed or resurrected to treat infections. Nevertheless, strains of MRSA with high-level resistance to vancomycin (VRSA) emerged [1]. Among Gram-negative bacteria, resistance of *Escherichia coli* to ciprofloxacin occurred within 10 hours in a microfluidic device with as few as 100 bacteria in the initial inoculation [3].

Since the early 1960 s, only four new classes of antibiotics have been introduced and the global antibiotics market is still dominated by antibiotic classes discovered half a century ago [4]. Apart from their high mortality rate, MRSA infections lead to additional health care costs, e.g. about $3 to 4 billion per year in the United States [4]. Bacteria can be alternatively killed by using methods such as autoclaving, UV and γ-radiation [5], hydrogen peroxide [6], [7], or chlorination [8], [9], but none of these methods can be safely applied in humans. Furthermore, the US National Academy of Sciences (NAS) together with twelve other national science academies recommended that combating antibiotic resistance is of particular importance for human health and this can be only accomplished by the discovery of new drugs that fight emerging multiresistant pathogens [10].

The photodynamic inactivation of bacteria (PIB) appears to be an additional and innovative modality to kill microorganisms. PIB is based on positively charged dye molecules (photosensitizers) that can attach to the negatively charged cell wall of the pathogens [11]–[13]. Then, the photosensitizer-loaded pathogens are exposed to visible light. The photosensitizer transfers the absorbed light energy to adjacent molecular oxygen leading to the generation of mainly singlet oxygen. This highly reactive molecule attacks bacterial cell wall components and hence causes an irreversible oxidative damage of the pathogens already during irradiation [14].

Using porphyrins or phenothiazins as photosensitizers, the research in the field of PIB has escalated showing first good results regarding inactivation of different pathogens [15]–[21]. However, many photosensitizers show low efficacy when simultaneously applied for different types of bacteria (Gram-positive, Gram-negative) or show moderate toxicity already without light. Both hampers clinical approval and hence broad application in clinical practice. In order to support the battle against the worldwide increase of infections with multiresistant bacteria, it is important that such molecules are effective and safe for applications in humans.

Nature offers many dye molecules like riboflavin that show a high ability to generate singlet oxygen when exposed to light [22]. This effect is known e.g. as disadvantageous reaction in food chemistry [23]. Flavin molecules are abundant redox cofactors and of key importance for many biological processes and should have a safe potential to be used in humans, food and other applications [24]. Therefore, we synthesized new flavin derivatives to create safe and effective photosensitizers against pathogens such as MRSA, enterohemorrhagic *Escherichia coli* (EHEC), multiresistant *Pseudomonas aeruginosa* and multiresistant *Acinetobacter baumannii*. The latter causes severe infections in the US military health care system [25].

## Materials and Methods

### Bacterial strains and growth conditions

Biochemical analysis and resistance testing of each bacterial strain were done with a Phoenix system (Becton, Dickinson and Company, Heidelberg, Germany), according to the guidelines of the Clinical and Laboratory Standards Institute (CLSI, Wayne, PA, USA). MRSA (ATCC BAA-44), multiresistant *Pseudomonas aeruginosa* (clinical isolate, University Hospital Regensburg), enterohemorrhagic *Escherichia coli* (EHEC) HUSECO41 (O104:H4; extended-spectrum β-lactamase (ESBL) producer) and multiresistant *Acinetobacter baumannii* (clinical isolate, University Hospital Regensburg) were used for photodynamic experiments *in vitro*. *Staphylococcus aureus* (ATCC 25923) was exemplarily used for singlet oxygen measurements in bacteria.

### Photosensitizer

**Figure 1:**
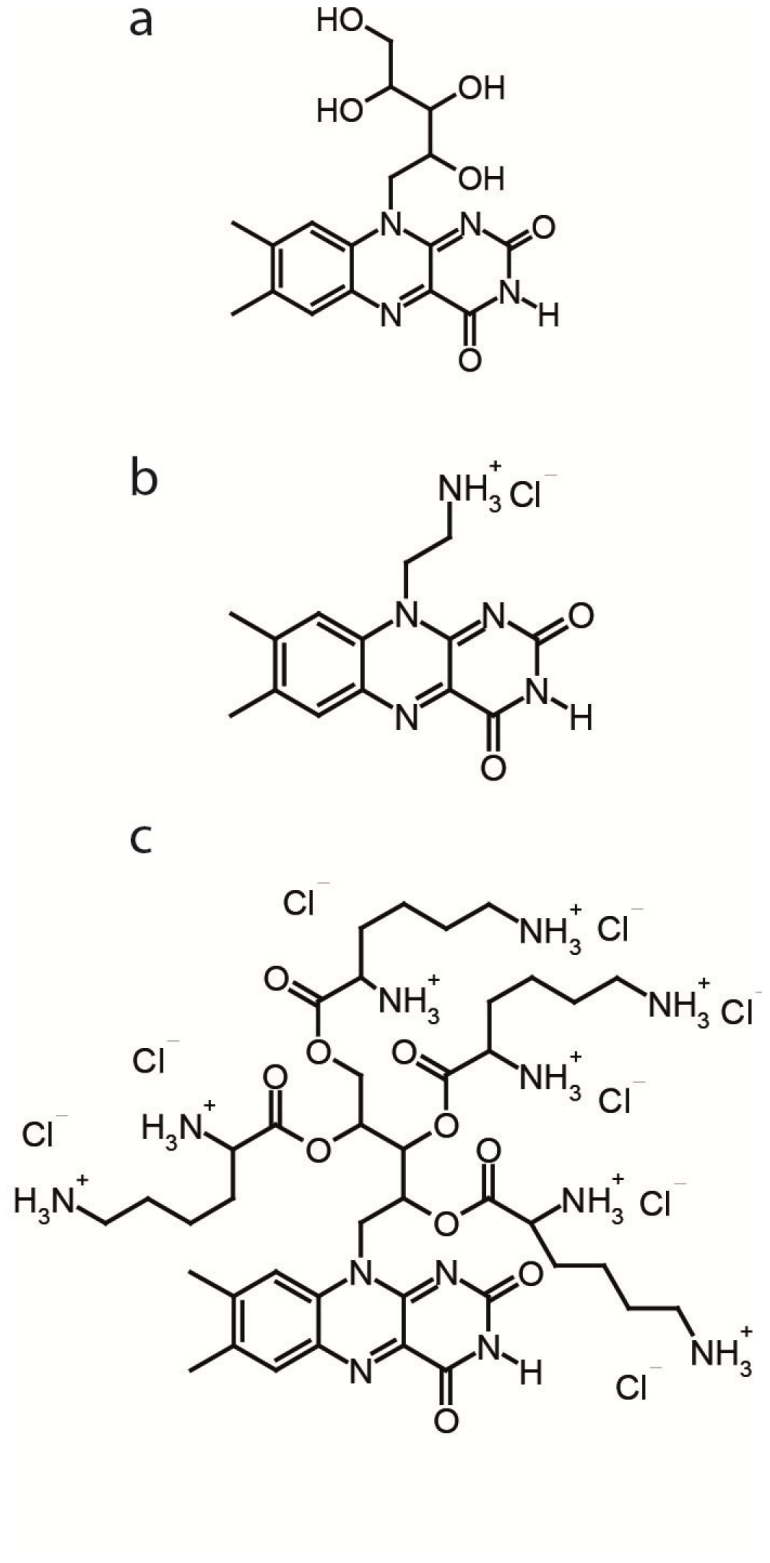
The chemical structure of riboflavin (a) is shown in comparison to the newly synthesized flavin molecules FLASH-01a (b), and FLASH-07a (c). FLASH-01a and FLASH-07a comprises one and eight positive charges, respectively.

The photosensitizer riboflavin (MW 376.2 g mol^−1^; purity>98% for biochemical application) was purchased from Sigma Aldrich (Steinheim, Germany). The photosensitizers FLASH-01a (single positive charge; MW 321.77 g mol^−1^; purity FLASH-01a-Hydrochloride:>98%, as determined by NMR spectroscopy in DMSO-d6 (Bruker Advance 400 MHz)) and FLASH-07a (eight positive charges; MW 1180.74 g mol^−1^; purity FLASH-07a-Hydrochloride:>95%, as determined by NMR spectroscopy in DMSO-d6 (Bruker Advance 600 MHz)) were synthesized at the Department of Chemistry, University of Regensburg, Germany (for more details please see Text S1). A basic flavin photosensitizer is prepared via directly attachment of one positive charge to the chromophore using a short alkyl chain linker. Therefore we prepared FLASH-01a as described in the literature [11] by the classical Kuhn synthesis protocol [26] (Fig. S1). In addition, we directly modified the alcohol groups of the ribose chain with lysine by Steglich esterification. After careful deprotection with hydrochloric acid in dry diethyl-ether, we obtained the new vitamin based photosensitizer FLASH-07a in sufficient amounts (Fig. S2–S4). The chemical structures of our new flavin photosensitizers are shown in Figure 1.

### Detection of singlet oxygen in solution

The quantum yield of singlet oxygen generation of FLASH-01a or FLASH-07a (each 50 µM) was determined in aqueous solution by comparison to a reference photosensitizer (5,10,15,20-tetrakis(1-methyl-4-pyridino)-porphyrin-tetra-(p-toluenesulfonate) using the method published by Baier *et al* [22].

### Detection of singlet oxygen in bacteria

*S. aureus* was aerobically cultivated overnight in Mueller-Hinton medium at 37°C. After cultivation, the suspension was centrifuged (3000 rpm, 10 min). The pellet was resuspended in Millipore water and was incubated with 500 µM FLASH-07a dissolved in Millipore water. After two washing steps (each 3000 rpm, 10 min), the suspension was transferred into a quartz glass cuvette. The singlet oxygen luminescence was detected time-resolved described earlier [27]. In addition, the luminescence signal was detected spectrally resolved at wavelengths of 1200, 1250, 1260, 1270 (emission maximum of singlet oxygen), 1280, 1300, 1350 and 1400 nm using appropriate interference filters in front of the photomultiplier [22]. For each measurement a fresh suspension was used. The number of laser pulses for excitation was 20.000; the output power was 182 mW and the excitation wavelength 450 nm.

### Light source and irradiation parameters

Bacterial suspensions were irradiated with a non-coherent light source (Waldmann PIB3000, Villingen-Schwenningen, Germany) emitting light from 380–600 nm. The emitted intensity was set to 50 mW cm^−^^2^; the radiant exposure of the light source was calculated by multiplying intensity with exposure time.

### Phototoxicity assay of bacteria

Approximately 10^8^ bacterial cells were placed into a 96-well microtiter plate and incubated with different concentrations of flavin derivatives (final concentrations 0/5/10/50/100 µmol L^−1^) for 10 sec in the dark. Subsequently after the incubation time, the suspensions were irradiated for different periods of time (Table 1 and 2). Upon irradiation, the survival of the bacteria was determined by counting the numbers of colony forming units (CFU) using the Miles, Misra and Irwin technique [28].

**Table 1:** Antimicrobial photodynamic efficacy of FLASH-01a.

Pathogen	Time [s]	Concentration [µM]	Log_10_-Reduction
**MRSA BAA-44**	30	5	3.1
	30	10	5.1
	30	50	5.1
	60	10	5.2
	**60**	**50**	**6.6**
***P. aeruginosa***	30	5	3.5
	30	10	4.7
	**30**	**50**	**6.8**
	**30**	**100**	**6.8**
**EHEC**	210	50	4.0
	240	10	4.6
	**240**	**50**	**6.5**
***A. baumannii***	120	50	4.4
	150	50	5.1
	180	10	5.8
	**180**	**50**	**6.6**
	**180**	**100**	**6.6**

The marked results (in bold) fulfill the FDA requirement of high level disinfection [30].

**Table 2:** Antimicrobial photodynamic efficacy of FLASH-07a.

Pathogen	Time [s]	Concentration [µM]	Log_10_-Reduction
**MRSA BAA-44**	10	50	3.9
	20	10	3.6
	**20**	**50**	**6.2**
	30	10	4.9
	**30**	**50**	**6.5**
	**30**	**100**	**6.5**
***P. aeruginosa***	30	10	5.3
	**30**	**50**	**6.8**
	60	5	4.1
	60	10	5.5
	**60**	**50**	**6.7**
**EHEC**	150	5	3.3
	**150**	**10**	**6.5**
	180	5	3.7
	180	10	4.5
	**180**	**50**	**6.6**
***A. baumannii***	60	10	3.7
	60	50	3.5
	90	10	5.7
	**90**	**50**	**6.7**

The marked results (in bold) fulfill the FDA requirement of high level disinfection [30].

#### Data analysis

All results are shown as means, including the corresponding standard deviations, which were calculated from the values of three independent experiments, each experiment was conducted in triplicate. The calculation was referred to untreated controls, which neither were incubated with photosensitizers nor irradiated.

### Eucaryotic cells and cell culture

Normal human epidermal keratinocytes (NHEKs) were purchased from ATCC (ATCC-PCS-200-010, American Type Culture Collection, Manassas, USA) and seeded into a T75 cell culture flask with 10 ml of Dermal Cell Basal Medium supplemented with Keratinocyte Growth Kit (both purchased from ATCC: PCS-200-030 and PCS-200-040, respectively). Cells were incubated at 37°C in a humidified atmosphere with 5% CO_2_ (v/v). The medium was replaced every two days. The NHEK cells were washed once with 10 ml PBS (Biochrom, Berlin, Germany) and removed from the flask bottom with 2 ml 0.1% trypsin-EDTA solution (Gibco Life Technologies, Eggenstein, Germany).

For PIB experiments the cells were seeded into 96-well microtiter plates (10.000 cells per well) and were incubated at 37°C and 5% CO_2_ overnight. On the next day, cells were incubated with different concentrations of flavin derivative solutions and illuminated with 50 mW cm^−2^. The flavin derivatives FLASH-01a and FLASH-07a were dissolved in DMEM medium (Dulbecco's Modified Eagle Medium, PAN Biotech Inc., Aidenbach, Germany) without serum and phenol red. After irradiation, the flavin solution was removed from each well and cells were incubated with 100 µL fresh Dermal Cell Basal Medium over night at 37°C and 5% CO_2_. Aliquots of treated and untreated cells (no photosensitizer with light, photosensitizer without light, and no photosensitizer/no light) were used as control values.

To evaluate the effects of irradiation with different concentrations of flavin derivatives on NHEK cells, the cell viability was directly estimated by 3-(4,5-dimethylthiazol-2-yl)-2,5-diphenyl-tetrazolium bromide (MTT) test as described by Mosmann [29].

### Transmission electron microscopy

The bacterial cell suspensions were treated as described in phototoxicity assay of bacteria. Ultrathin sections (80 nm) were cut examined in a LEO912AB transmission electron microscope (Zeiss, Oberkochen, Germany) operating at 100 kV. Images were recorded using OSIS-Software iTEM (Olympus Soft Imaging Solutions, Münster, Germany).

## Results

### Singlet oxygen generation of the new photosensitizer molecules

**Figure 2:**
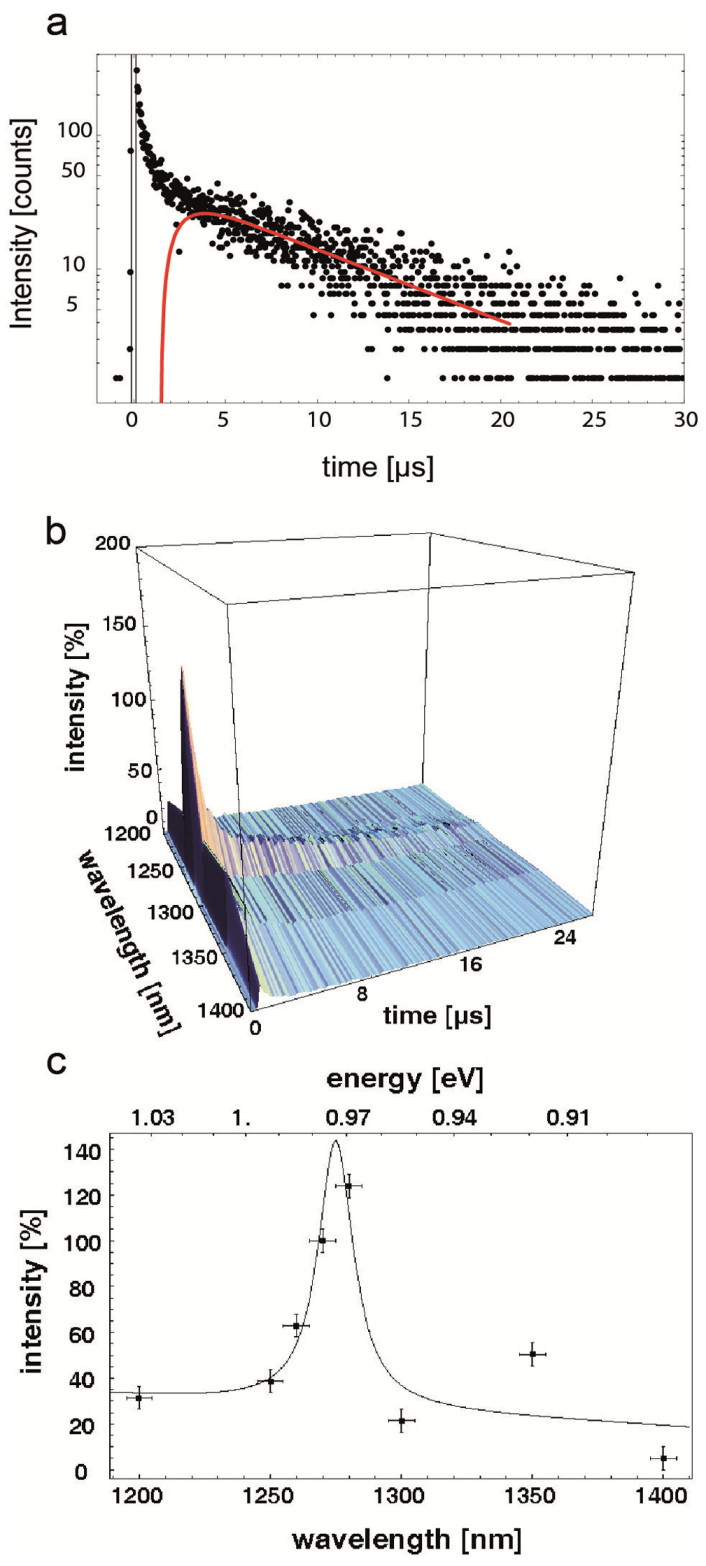
a. Time resolved luminescence signal of singlet oxygen. The red fit line exemplarily shows a time resolved luminescence signal of singlet oxygen in *S. aureus* suspensions generated by FLASH-07a. b. Fingerprint of singlet oxygen. The time resolved (a) and spectrally resolved (c) luminescence signals of singlet oxygen generated by FLASH-07a can be combined to achieve a clear fingerprint of singlet oxygen generation. c. Spectrally resolved luminescence signal of singlet oxygen. Singlet oxygen signal of a *S. aureus* suspension incubated with FLASH-07a. The fitting of the measured values (black line) shows a clear peak at about 1270 nm indicating that singlet oxygen is generated.

Singlet oxygen was directly detected by its luminescence at about 1270 nm using a highly sensitive photomultiplier technology [14]. Both newly synthesized photosensitizers, FLASH-01a and FLASH-07a, generated singlet oxygen with a quantum yield of 0.75±0.05 and 0.78±0.05, respectively. When *S. aureus* was exemplarily incubated with FLASH-07a and after two washing steps using Millipore water, a clear spectrally and time resolved luminescence signal of singlet oxygen was detected (Fig. 2). This provides direct evidence that singlet oxygen is generated close or inside *S. aureus* and confirms the attachment of the photosensitizer to the bacteria.

### Killing of Gram-positive multiresistant bacteria

**Figure 3:**
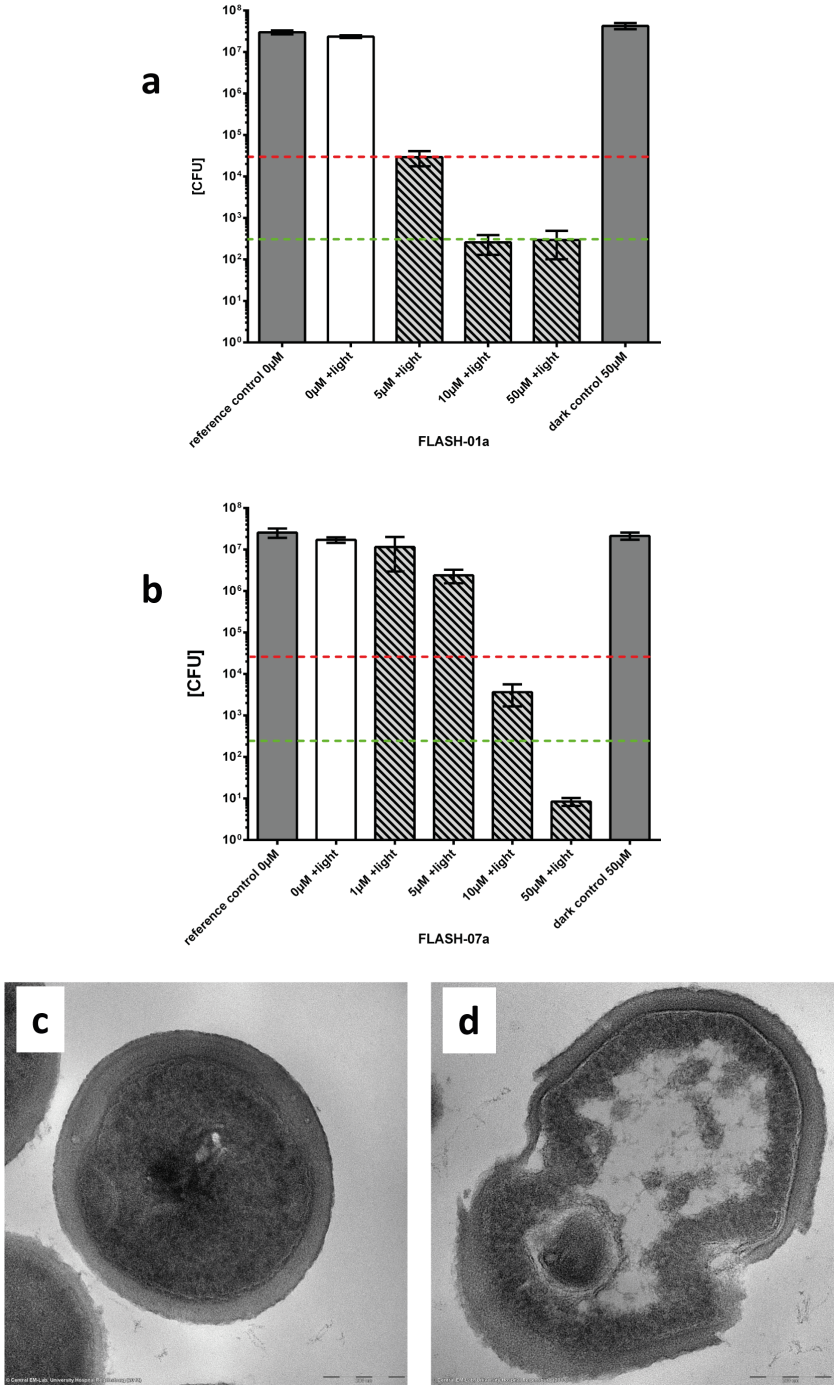
Survival of MRSA strain BAA-44. Bacteria were incubated with FLASH-01a or FLASH-07a for 10 seconds in the dark. Suspensions were subsequently irradiated with 50 mW cm^−2^ for 30 (1.5 J cm^−2^; Fig. 3a) or 20 seconds (1 J cm^−2^; Fig. 3b) (hatched bars). Controls: bacteria alone (reference control 0 µM) or incubated with FLASH-01a or −07a only (dark control 50 µM), but not irradiated (grey bars); bacteria alone, but irradiated (0 µM+light; white bar). Bars represent the means including the corresponding standard deviations of three independent experiments. Values on or below the dotted horizontal line represent ≧99.9% (red) or ≧99.999% (green) efficacy of bacteria killing, which was referred to the untreated controls (bacteria alone). TEM images show MRSA before (Fig. 3c) and after (Fig. 3d) PIB treatment (50 µM FLASH-07a, 50 mW cm^−2^, irradiation period 60 s).

Methicillin resistant *Staphylococcus aureus* (MRSA) is a causative agent of skin and soft tissue infections. The MRSA strain BAA-44 was incubated with different concentrations of FLASH-01a or FLASH-07a for 10 seconds and was subsequently irradiated with 50 mW cm^−2^ for 20 (1 J cm^−2^) or 30 seconds (1.5 J cm^−2^). Here bacterial solutions incubated with flavin photosensitizers were not washed due to a prospective topical application *in vivo*. A FLASH-01a concentration of 10 µmol L^−1^ resulted in a bacterial killing of ≧5 log_10_ orders (≧99.999%) (Fig. 3a).

The most efficient reduction of MRSA was achieved when bacteria were incubated with 50 µmol L^−1^ of FLASH-07a and irradiated with a total radiant exposure of 1.0 J cm^−2^ (Fig. 3b). This PIB treatment results in a killing rate of more than 6 log_10_ orders (>99.9999%). A further increase of FLASH-01a/−07a concentrations and illumination time did not enhance the killing effect (Tables 1+2). Incubation of MRSA with light but no photosensitizer (light control) or photosensitizer but no light (dark control) or no light and no photosensitizer (reference control) revealed no bactericidal effect within experimental accuracy. Thus, only the combination of photosensitizer and light can efficiently kill the bacteria. In addition, no toxicity of both flavin derivatives alone (dark control) could be observed for concentrations up to 500 µmol L^−1^ (data not shown). This is a first and impressive proof of the safety of our new flavin photosensitizers.

### Killing of Gram-negative multiresistant bacteria

In our study, we focused on three important multiresistant Gram-negative strains, multiresistant *Pseudomonas aeruginosa,* enterohemorrhagic *Escherichia coli* (EHEC), and multiresistant *Acinetobacter baumannii*.

*P. aeruginosa* was incubated with different concentrations ranging from 5 µmol L^−1^ to 100 µmol L^−1^ of FLASH-01a for 10 seconds and was subsequently irradiated with 50 mW cm^−2^ for 30 seconds (1.5 J cm^−2^). The reduction of bacterial survival ranged from 3.5 log_10_ orders (≧99.9%) to ≧6 log_10_ orders, which is equivalent to a killing efficacy of 99.9999% of *P. aeruginosa* cells (Table 1). The use of FLASH-07a yielded comparable results (Table 2). Similar bactericidal effects could be observed when PIB was applied in *A. baumannii* or EHEC. However, irradiation time and hence the applied light energy for killing of *A. baumannii* or EHEC was higher as compared to MRSA or *P. aeruginosa*.

Irradiation of EHEC suspensions for 240 seconds with 50 µmol L^−1^ of FLASH-01a showed a reduction rate in cell viability of ≧6 log_10_ steps (Table 1). FLASH-07a was as effective as FLASH-01a when using the same concentration (50 µmol L^−1^) but the antibacterial effect was already achieved after 180 seconds of FLASH-07a irradiation (Table 2).

Irradiation of multiresistant *A. baumannii* for 180 seconds with 50 µmol L^−1^ of FLASH-01a showed a reduction rate in cell viability of ≧6 log_10_ steps (≧99.9999%) (Table 1). When *A. baumannii* was incubated with 50 µmol L^−1^ of FLASH-07a, the killing rate of ≧6 log_10_ steps was already received after 90 seconds (Table 2). These results show the high potential of the FLASH-07a derivative. When compared to FLASH-01a, the increased number of positive charges on almost all sides of the FLASH-07a molecule might have enhanced the attachment of the photosensitizer to the surface of bacteria. In addition, PIB with both photosensitizers provides a high killing efficacy (>6 log_10_ orders) according to the FDA requirements for high level disinfection [30].

### Transmission electron microscopy imaging of flavin treated MRSA

Images were exemplarily recorded of MRSA treated with FLASH-07a using transmission electron microscopy (Fig. 3). After photodynamic treatment, morphological changes in particular of the cytoplasmic lipid membrane were observed (Fig. 3d). The most pronounced effect is indicated as mesosome-like structures that are formed upon photodynamic treatment. This observation was found in almost all treated cells, whereas control cells did not show this effect (Fig. 3c). Further, some of the cells showed a disruption of the peptidoglycan layer, which might enable an easier penetration of the photosensitizer into the interior of the bacterial cell during irradiation. These images visualize the killing effect of bacteria by means of PIB.

### Toxicity of new flavin derivatives in eukaryotic cells

**Figure 4:**
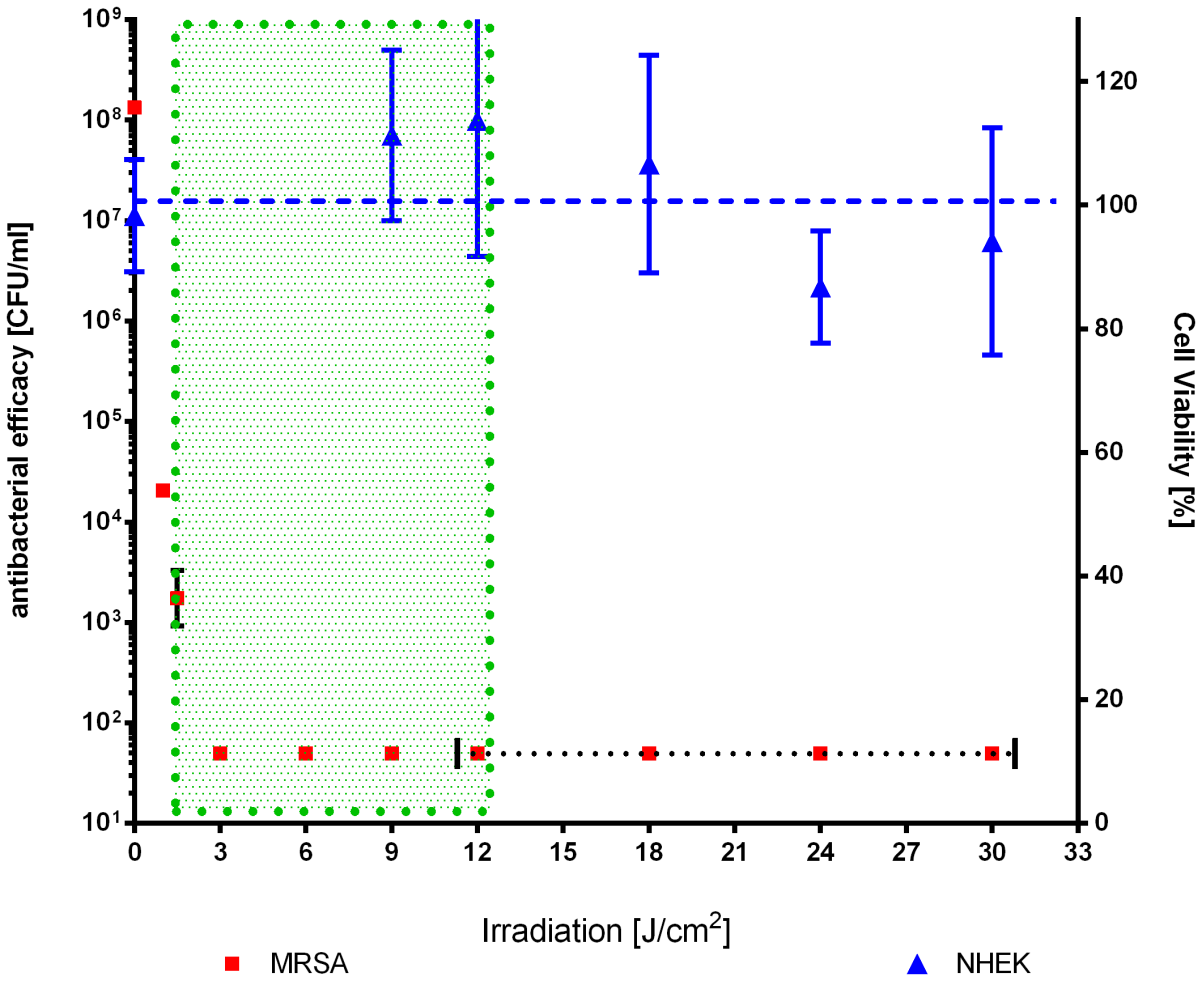
Photodynamic toxicities of FLASH-07a for NHEKs and MRSA at different light intensities. NHEKs (blue triangles) and MRSA (red squares) were incubated with 10 µM of FLASH-07a for 10 seconds. After radiation with different light intensities (MRSA: up to 9 J/cm^2^; NHEK: from 9–30 J/cm^2^) viability of NHEK and MRSA was determined 24 h later. Phototoxicity was determined by MTT assay or CFU assay for NHEK or MRSA, respectively. Values represent the means and standard deviations of three independent experiments. Blue dashed line: regression line of NHEK viability. Green dotted box: Photodynamic conditions were MRSA can be efficiently inactivated (>5 log_10_ reduction) without NHEK cell killing (‘therapeutic window’). Dotted horizontal black line: CFU of MRSA below detection limit.

The cell toxicity of FLASH-01a and FLASH-07a was tested against normal human epidermal keratinocytes (NHEKs). The cells were incubated with FLASH-01a or FLASH-07a with concentrations up to 100 µM and irradiated with the same light parameters as used for PIB (Table 3). The results of the MTT assay clearly showed that cell viability was not affected by both photosensitizers for radiant exposures up to 9 J cm^−^^2^ (FLASH-07a) or 12 J cm^−^^2^ (FLASH-01a). In order to confirm a therapeutic window the photodynamic efficacy of FLASH-07a against MRSA is plotted versus the cell viability of NHEKs (Fig. 4). Figure 4 shows the range of different used light intensities (up to 30 J cm^−2^) at a given photosensitizer concentration of 10 µM for efficiently killing of MRSA (>5 log_10_ reduction at 9 J cm^−2^; green dotted box) while NHEKs were not affected under these conditions.

**Table 3:** Photodynamic effect on human keratinocytes – NHEK cell viability [%].

**FLASH-01a**	**0 µM**	**10 µM**	**50 µM**	**100 µM**
+ irradiation (12 J cm^−2^)	100^a^	105±18	98±17	98±21
− irradiation (dark control)	100^a^	96±6	95±11	93±12
**FLASH-07a**	**0 µM**	**10 µM**	**50 µM**	**100 µM**
+ irradiation (9 J cm^−2^)	100^a^	105±14	102±13	101±17
− irradiation (dark control)	100^a^	95±7	99±12	95±17

a Cell viability was normalized to corresponding untreated controls (no light, no photosensitizer).

## Discussion

Many vitamins can generate singlet oxygen particularly when exposed to short wavelength radiation and therefore appear to be suitable for PIB [31], [32]. To avoid hazard ultraviolet radiation in PIB, vitamins of the B group seemed to be the most suitable molecules showing a sufficient absorption of visible light. Vitamins like riboflavin (B2), niacin (B3) or pyridoxine (B6) play an important role in the cell metabolism process and can be considered safe when administered to humans. The maximum level of daily nutrient intake in adults that is likely to pose no risk of adverse effects for e.g. pyridoxine is 100 mg [33]. Among the B vitamins riboflavin exhibits the highest quantum yield of singlet oxygen generation (Φ_Δ_ = 0.54) [22], [34], which should be in principle sufficient to inactivate microorganisms via PIB. However, the use of light activated riboflavin showed hardly any bacterial killing effects [35], [36]. This is not surprising because such uncharged molecules cannot attach to the cell wall of bacteria and hence yield no or minimal antimicrobial effect [37].

Upon light irradiation, the photosensitizer converts the absorbed light energy to reactive oxygen species (ROS) like singlet oxygen. To destroy bacteria via oxidation, singlet oxygen has to reach bacteria by diffusion within its short lifetime of a few microseconds. The corresponding diffusion length of singlet oxygen is less than one micrometer [14]. To achieve a high efficacy of bacteria killing, singlet oxygen has to be generated directly at the cell wall or inside the cell. Thus, it is a major prerequisite for photosensitizers that the molecules attach to the cell wall of bacteria at least.

Beside singlet oxygen, other ROS like superoxide anions could contribute to the killing mechanism of the PIB treatment. The singlet oxygen quantum yields of our flavin derivatives were 0.75±0.05 (FLASH-01a) and 0.78±0.05 (FLASH-07a), respectively. Thus, the remaining light energy is split into internal conversion of the photosensitizer (fluorescence, heat emission) and type I ROS like superoxide anions. Therefore, it is likely that the main reacting ROS is singlet oxygen and not type I ROS like superoxide anions. The PIB mediated damage of DNA is controversially discussed. *Deinococcus radiodurans* is a Gram-positive bacterium that is well known to be resistant to UV and γ-radiation due to a very effective DNA repair mechanism. Schaefer *et al*. showed that *D. radiodurans* was even more susceptible to PIB treatment than *E. coli* suggesting that DNA damage could not be the primary mechanism of cell killing mediated by PIB [38].

To open the door for PIB into many fields of application in medicine and even in food industry, the photosensitizer has to fulfill a series of requirements. The molecule should be safe, positively charged, photostable, should absorb light in the visible spectrum and effectively convert the absorbed light energy to singlet oxygen. The killing rate should be effective at low photosensitizer concentrations and small radiant exposures of less than 10 J cm^−^^2^. It is additionally of importance that the killing effect is preferably independent of the type of the attacked bacteria. Both new flavin-based photosensitizers killed MRSA as much as *S. aureus* after PIB treatment, indicating that the mechanism of methicillin resistance did not affect the photosensitizer attachment or uptake to this bacteria species. Such observation is important, because *S. aureus* can develop resistance to antibiotics or biocides due to a limited penetration, as shown for vancomycin-intermediate-resistant *S. aureus* strains, which produce markedly thicker peptidoglycan layers [39]. As a consequence such cell wall changes like an increased thickness of the cell wall or different patterns of cross-linking of the peptidoglycan layers did not lead to a reduced efficacy of PIB as compared to the use of the last line of defence antibiotic vancomycin.

Furthermore, we focused here on three important, multiresistant Gram-negative strains, namely enterohemorrhagic *E. coli* (EHEC), multiresistant *P. aeruginosa* and multiresistant *A. baumannii*. EHEC infections can cause bloody diarrhea, hemorrhagic colitis and gastroenteritis with often severe complications like the hemolytic uremic syndrome (HUS). A novel Shiga-toxin producing *E. coli* strain O104:H4 caused a large outbreak of infections in Germany in 2011 [40]. 3842 cases of infections were reported, whereof 855 patients were suffering from HUS disease and finally 53 people died [41]. In addition, other countries in Europe reported the O104:H4 outbreak. In addition, the most common serotype O104:H7 caused severe HUS associated infections in North America [40].

*P. aeruginosa* is one of the most important nosocomial pathogens that cause severe wound infections in burn patients [42], chronic lung infections preferably in cystic fibrosis patients [43] or other hospital acquired infections like urinary tract infections due to formation of biofilms in catheters [44]. *P. aeruginosa* displays a high ability to resist antibiotics intrinsically [45] but multidrug resistance occurs even by acquisition of resistance genes, over-expression of efflux pumps, decreased expression of porins or mutations. *P. aeruginosa* is even able to metabolize sodium dodecyl sulfate (SDS), a biocide detergent to all known bacteria so far [46].

The rapid emergence of multiresistant *A. baumannii* isolates, which exhibit resistance to the most available antibiotics during the last decade, is a worrying evolution. This bacterium belongs to the predominant pathogens in hospitals and especially at military medical facilities in the Iraq/Kuwait region during Operation Iraqi Freedom (OIF) and in Afghanistan during Operation Enduring Freedom (OEF) [25].

When applying usual photosensitizers, Gram-negative bacteria are more resistant to PIB than Gram-positive bacteria due to their cell wall structure [47]. Although the cell wall of Gram-positive bacteria is composed of up to 100 peptidoglycan layers (thickness ∼40–80 nm), the structure is not protective against penetration of positively charged PS. In contrast, the additional outer membrane of Gram-negative bacteria hampers the attachment and uptake of photosensitizers and represents a very potent diffusion barrier. Nevertheless, the use of our new flavin photosensitizers yielded a very efficient and fast killing of all three multiresistant Gram-negative bacteria. Many types of bacteria have learnt to overcome or bypass the specific damage mechanisms caused by antibiotics during the past decades. In contrast to that, the use of FLASH-01a or FLASH-07a killed both, multiresistant Gram-positive and Gram-negative bacteria with an outstanding antimicrobial efficacy (>6 log_10_ reduction). According to the FDA guidelines, the killing efficacy of >99.999% represents a high level disinfection [30]. Moreover, the attachment of FLASH-01a and −07a photosensitizers to bacteria is simply based on electrostatic interaction of the positively charged photosensitizer with the negatively charged surface of bacteria. Upon irradiation, the subsequent oxidative attack of singlet oxygen targets all double bounds in biomolecules of bacteria such as lipids and proteins. Thus, it seems to be very unlikely that PIB causes any new resistance of bacteria. In addition, no resistance of photodynamically treated bacteria has been reported so far [48], [49].

PIB is not intended to replace antibiotics but to support them to gain time for the development of new antibiotics. PIB should be suitable for an efficient killing of multiresistant bacteria on surfaces such as skin and mucosa, maybe even in wounds and soft tissue. Efficient decolonization of multiresistant bacteria should help to avoid life-threatening incidents in humans. Therefore, an important goal in the investigation of photosensitization processes in antimicrobial PIB is to elucidate a therapeutic window, in which bacteria are killed without harming the surrounding tissue such as the skin. The results of the present study showed that the photosensitization of bacteria with FLASH-01a or FLASH-07a yielded an inactivation of 6 log_10_ orders at least with a radiant exposure of 9 J cm^−^^2^. In contrast, cell viability of human keratinocytes was not affected by both photosensitizers for radiant exposures up to 30 J cm^−^^2^. Thus, PIB with both flavin derivatives can be considered safe in humans showing a great potential of bacterial killing without harming the adjacent tissue (‘therapeutic window’). Recently Maisch *et al*. could also demonstrate a therapeutic window *ex vivo* using a new developed porphyrin derivative XF73 [50].

Our new photosensitizers are vitamin derivatives and demonstrated a photodynamic killing efficacy of >5 log_10_ orders upon light activation within a few seconds against different types of multiresistant bacteria. Under these conditions, human keratinocytes are still alive which is an important prerequisite to use it *in vivo*. In a next ongoing study, we would like to develop an appropriate flavin derivative formulation to test the PIB efficacy *in vivo* using a burn wound mice model.

## Supporting Information

Figure S1 **Synthesis of FLASH-01a** (**2**); Conditions: (a) MeOH, HOAc, Pd/C, H_2_, RT, 12 h, not isolated, quant.; (b) alloxan monohydrate, boric acid, MeOH, RT, in the dark, nitrogen atmosphere, 1d, 72%; (c) DCM, HCl in Et_2_O, RT, moisture protection, in the dark, 4 h, 93%.
(TIF)

Figure S2 **Synthesis of FLASH-07a** (**3**); Conditions: (a) DMF, boc-Lys(boc)-OH, DMAP, DCC, RT, in the dark, overnight, 49%; (b) DCM, HCl in Et_2_O, RT, in the dark, moisture protection, 4 h, 86%.
(TIF)

Figure S3 **Boc-protected FLASH-07a.** 2,6-Bis-tert-butoxycarbonylamino-hexanoic acid 2,3,4-tris-(2,6-Bis-tert-butoxycarbonylamino-hexanoxy)-5-(7,8-dimethyl-2,4-dioxo-3,4-dihydro-2H-benzo[g]pteridin-10-yl)-pentyl ester.
(TIF)

Figure S4 **Deprotected flavin photosensitizer FLASH-07a.** 2,6-Bis-amino-hexanoic acid 2,3,4-tris-(2,6-bis-amino-hexanoxy)-5-(7,8-dimethyl-2,4-dioxo-3,4-dihydro-2H-benzo[g]pteridin-10-yl)-pentyl esteroctahydrochloride.
(TIF)

Text S1 **Chemical synthesis of flavin photosensitizers.**
(DOCX)
