# Supplementary figures and images for: Fast and Effective Photodynamic Inactivation of Multiresistant Bacteria by Cationic Riboflavin Derivatives
Source: PLoS One. 2014 Dec 3;9(12):e111792. doi: 10.1371/journal.pone.0111792 (PMC4254278; doi:10.1371/journal.pone.0111792)

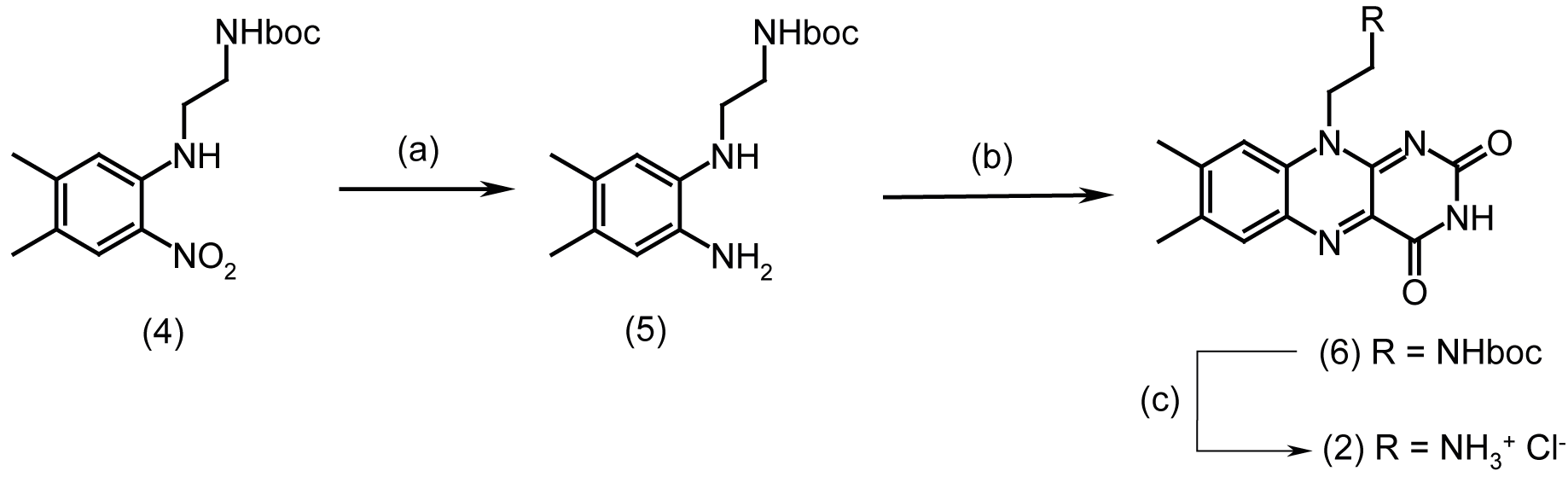

Supplement: Figure S1 — Synthesis of FLASH-01a (2); Conditions: (a) MeOH, HOAc, Pd/C, H2, RT, 12 h, not isolated, quant.; (b) alloxan monohydrate, boric acid, MeOH, RT, in the dark, nitrogen atmosphere, 1d, 72%; (c) DCM, HCl in Et2O, RT, moisture protection, in the dark, 4 h, 93%. (TIF) [file pone.0111792.s001.tif]

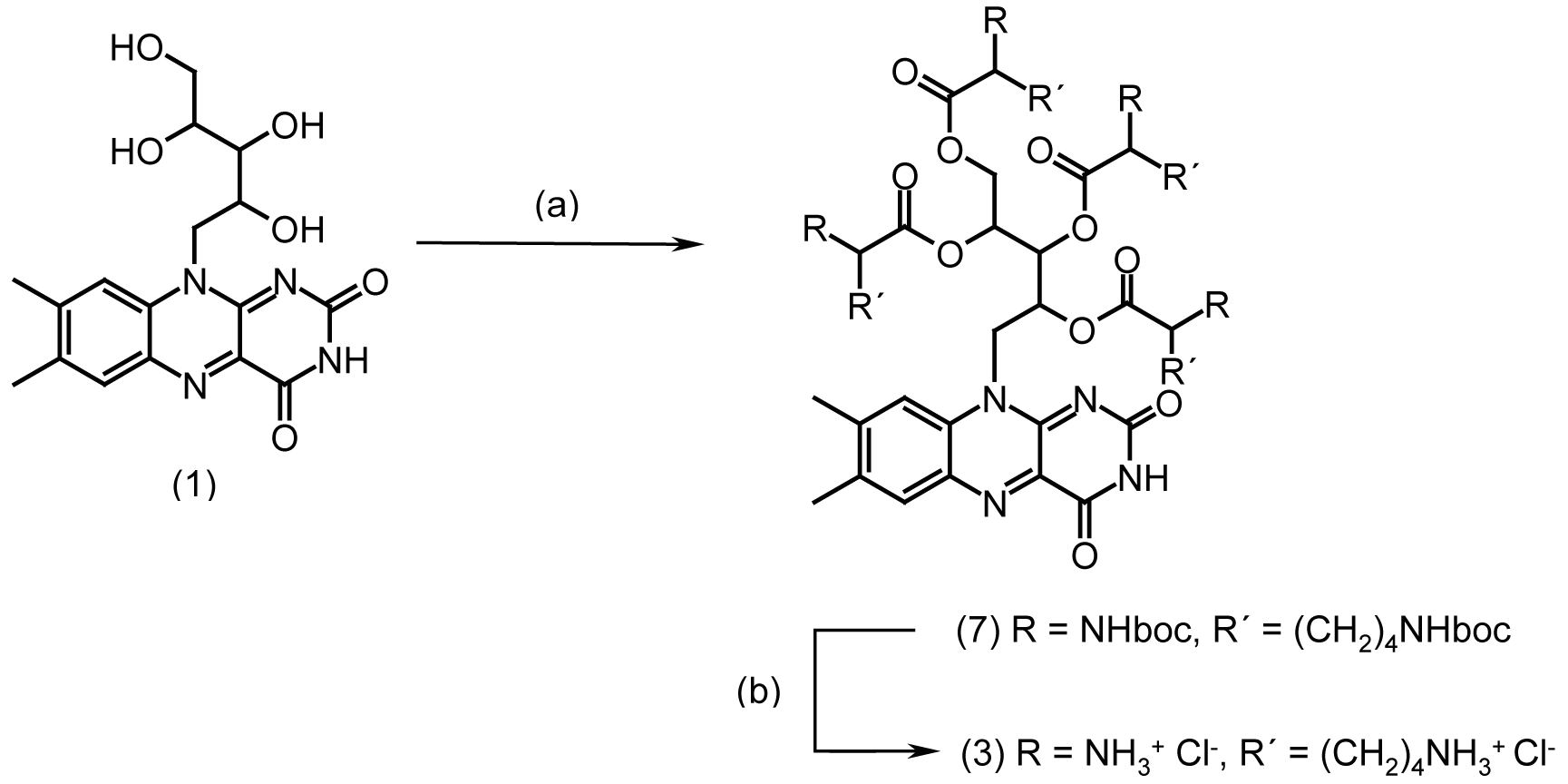

Supplement: Figure S2 — Synthesis of FLASH-07a (3); Conditions: (a) DMF, boc-Lys(boc)-OH, DMAP, DCC, RT, in the dark, overnight, 49%; (b) DCM, HCl in Et2O, RT, in the dark, moisture protection, 4 h, 86%. (TIF) [file pone.0111792.s002.tif]

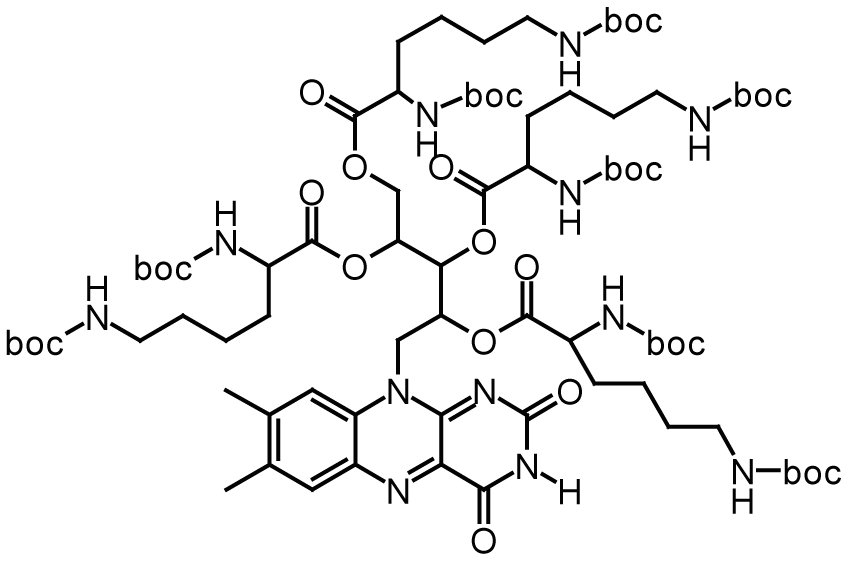

Supplement: Figure S3 — Boc-protected FLASH-07a. 2,6-Bis-tert-butoxycarbonylamino-hexanoic acid 2,3,4-tris-(2,6-Bis-tert-butoxycarbonylamino-hexanoxy)-5-(7,8-dimethyl-2,4-dioxo-3,4-dihydro-2H-benzo[g]pteridin-10-yl)-pentyl ester. (TIF) [file pone.0111792.s003.tif]

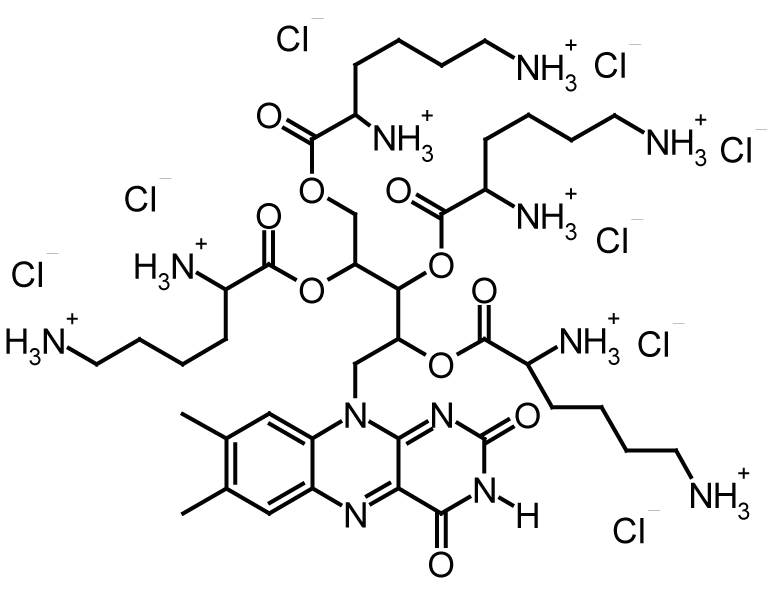

Supplement: Figure S4 — Deprotected flavin photosensitizer FLASH-07a. 2,6-Bis-amino-hexanoic acid 2,3,4-tris-(2,6-bis-amino-hexanoxy)-5-(7,8-dimethyl-2,4-dioxo-3,4-dihydro-2H-benzo[g]pteridin-10-yl)-pentyl esteroctahydrochloride. (TIF) [file pone.0111792.s004.tif]
