# Supplementary material for: Fast and Effective Photodynamic Inactivation of Multiresistant Bacteria by Cationic Riboflavin Derivatives
Source: PLoS One. 2014 Dec 3;9(12):e111792. doi: 10.1371/journal.pone.0111792 (PMC4254278; doi:10.1371/journal.pone.0111792)
Supplement: Text S1 — Chemical synthesis of flavin photosensitizers. (DOCX) [file pone.0111792.s005.docx]

**Text S1: Chemical synthesis of flavin photosensitizers**

1. **Synthesis and Characterization of the compounds**

Analytical characterization of the synthesized compounds was done by common methods. Melting Points were determined on Büchi SMP or a Lambda PhotometricsOptiMelt MPA 100 and are uncorrected. IR spectra were recorded with a Bio-Rad FT-IR Excalibur FTS 3000 equipped with a Specac*Golden Gate* Diamond Single Reflection ATR-System. Absorption spectra were recorded on a Varian Cary BIO 50 UV/VIS/NIR spectro­meter with temperature control using 1 cm quartz cuvettes (Hellma) and Uvasol solvents (Merck, Baker or Acros). Fluorescence measurements were performed with UV-grade solvents (Baker or Merck) in 1 cm quartz cuvettes (Hellma) and recorded on a Varian ‘Cary Eclipse’ fluorescence spectrophotometer with temperature control. Electro spray mass spectra were performed on a Finnigan MAT TSQ 7000 ESI-spectrometer. Other mass spectra were recorded on Varian CH-5 (EI), Finnigan MAT 95 (CI; FAB and FD); Xenon serves as the ionization gas for FAB. NMR spectra were recorded on BrukerAvance 600 (^1^H: 600.1 MHz, ^13^C: 150.1 MHz, T = 300 K), BrukerAvance 400 (^1^H: 400.1 MHz, ^13^C: 100.6 MHz, T = 300 K) or BrukerAvance 300 (^1^H: 300.1 MHz, ^13^C: 75.5 MHz, T = 300 K) relative to external standards. NMR spectra were recorded in CDCl_3_ at 300 MHz (^1^H) or 75 MHz (^13^C) unless stated otherwise.

Characterization of the signals: s = singlet, d = doublet, t = triplet, q = quartet, m = multiplet, bs = broad singlet, dd = double doublet, dt = double triplet, ddd = double double doublet. Integration is determined as the relative number of atoms, the coupling constants are given in hertz [Hz]. The multiplicity of the carbon atoms is given as (+) = CH_3_ or CH, (-) = CH_2_ and (C_quat_) for quaternary carbon atoms. Error of reported values: chemical shift: 0.01 ppm for ^1^H-NMR, 0.1 ppm for ^13^C-NMR and 0.1 Hz for coupling constants. The solvent used is reported for each spectrum. Analytical TLC plates (silica gel 60 F_254_) and silica gel 60 (70-230 or 230-400 mesh) were used for chromatographic separations. Visualization of the spots was done by UV light and/or staining with ninhydrin in ethanol. PE means petrol ether with a boiling range of 70 - 90^o^ C. All other solvents and chemicals were of reagent grade and used without further purification. Riboflavin was purchased from Sigma Aldrich in a purity of > 98% and was used as received. Boc-Lys(boc)-OH * DCHA was purchased from Bachem in a purity of > 99%. It was washed with 5% KHSO_4(aq)_ to give the free acid in quantitative yield, which was used without further purification.

The porphyrin based photosensitizer TMPyP (5,10,15,20-tetrakis(1-methyl-4-pyridino)-porphyrin-tetra-(p-toluenesulfonate) was used as the reference photosensitizer (purity 97% Sigma-Aldrich, Steinheim, Germany) and.

1. **Preparation of FLASH-01a** *(7,8-dimethyl-10-(2′-aminoethyl)-10H-benzo[g]pteridine-2,4-dione hydrochloride)*

The following compounds were prepared after literature known procedures [[51](#_ENREF_51)] (Fig. S1).

1. **Preparation of FLASH-07a**

Synthesis of FLASH-07a: Fig. S2

Boc-protected FLASH-07a: Fig. S3

Deprotected flavin photosensitizer FLASH-07a: Fig. S4

Riboflavin (1.13 g, 3.0 mmol) was suspended in dry DMF (120 mL) at room temperature. The boc-protected amino acid (10.0 mmol, 2.5 eq per OH group) was added, followed by addition of DMAP (0.49 g, 4 mmol). After stirring for 5 min at room temperature, DCC (3.71 g, 18 mmol, 1.5 eq per OH group) was added in small portions. The reaction mixture was stirred over night at room temperature under light protection in nitrogen atmosphere. Another portion of DCC (2.47 g, 12 mmol, 1.0 eq per OH group) was added and stirring was continued for 12 h.

The reaction mixture was diluted with ethyl acetate (300 mL), washed with saturated aqueous ammonium chloride solution (100 mL), water (100 mL) and brine (100 mL). The organic phase was separated, dried over MgSO_4_ and filtered. The solvent was evaporated under reduced pressure in the dark. The residue was taken up in ethyl acetate (30 mL). The solution was filtered to remove any dicyclohexylurea. The filtrate was evaporated and the residue was purified by column chromatography with silica gel using a gradient of dichloromethane/ethanol 50:1 🡪 20:1 and with dichloromethane/ethanol 20:1.

Yellow gas (49% yield). Light sensitive compound.

**^1^H-NMR** (300 MHz, CDCl_3_): δ [ppm] = 8.91 (br s, 1H, NH), 7.95 (s, 1H), 7.72 (s, 1H), 5.93 (bs, 1H) 5.82 (br s, 1H), 5.64 – 5.42 (m, 2H), 5.28 - 4.85 (m, 3H), 4.65 (m, 1 H), 4.40 - 4.20 (m, 3H), 3.08 (m, 8H), 2.53 (s, 3H), 2.40 (s, 3H), 1.95 – 1.65 (m, 8H), 1.62 – 1.20 (m, 16H), 1.44 (s, 18H), 1.43 (s, 36H), 1.41 (s, 18H), further signals were not detectable; - **MS** (ESI, MeOH) m/z: 1690.2 [M + H^+^]^+^ (100 %), 1707.3 [M + NH_4_^+^]^+^ (64 %), 1711.9 [M + Na^+^]^+^ (6 %), 845.8 [M + 2H^+^]^2+^ (87 %).

The deprotection of the boc-protected compound was done by a standard protocol using dichloromethane and HCl in diethylether under dry conditions [[52](#_ENREF_52)].

Orange, hygroscopic powder (86% yield). Light sensitive compound.

**MS** (ESI, MeOH) m/z: 889.9 [M + H^+^]^+^ (9 %), 445.3 [M + 2H^+^]^2+^ (100 %), 297.1 [M + 3H^+^]^3+^ (62 %).
